# GPU Accelerated Hybrid Particle‐Field Molecular Dynamics: Multi‐Node/Multi‐GPU Implementation and Large‐Scale Benchmarks of the OCCAM Code

**DOI:** 10.1002/jcc.70126

**Published:** 2025-05-14

**Authors:** Rosario Esposito, Giuseppe Mensitieri, You‐Liang Zhou, Zhong‐Yuan Lu, Ying Zhao, Toshihiro Kawakatsu, Giuseppe Milano

**Affiliations:** ^1^ Department of Chemical, Materials and Production Engineering University of Naples Federico II Napoli Italy; ^2^ State Key Laboratory of Supramolecular Structure and Materials, College of Chemistry Jilin University Changchun China; ^3^ School of Physics and Materials Engineering Dalian Minzu University Dalian China; ^4^ Department of Physics Tohoku University Sendai Japan

**Keywords:** coarse‐graining, GPU‐accelerated molecular dynamics, hybrid particle‐field method, large‐scale simulations

## Abstract

A parallelization strategy for hybrid particle‐field molecular dynamics (hPF‐MD) simulations on multi‐node multi‐GPU architectures is proposed. Two design principles have been followed to achieve a massively parallel version of the OCCAM code for distributed GPU computing: performing all the computations only on GPUs, minimizing data exchange between CPU and GPUs, and among GPUs. The hPF‐MD scheme is particularly suitable to develop a GPU‐resident and low data exchange code. Comparison of performances obtained using the previous multi‐CPU code with the proposed multi‐node multi‐GPU version are reported. Several non‐trivial issues to enable applications for systems of considerable sizes, including large input files handling and memory occupation, have been addressed. Large‐scale benchmarks of hPF‐MD simulations for system sizes up to 10 billion particles are presented. Performances obtained using a moderate quantity of computational resources highlight the feasibility of hPF‐MD simulations in systematic studies of large‐scale multibillion particle systems. This opens the possibility to perform systematic/routine studies and to reveal new molecular insights for problems on scales previously inaccessible to molecular simulations.

## Graphical abstract

A GPU‐resident parallelization of OCCAM code, suitable for multi‐node architectures, is proposed for hybrid particle‐field molecular dynamics. During simulations, excluding initialization steps, no further data exchanges between the CPU and GPU are performed, and communication operates only among GPUs. The proposed implementation allows multibillion‐particle simulations, allocating moderate amounts of resources.

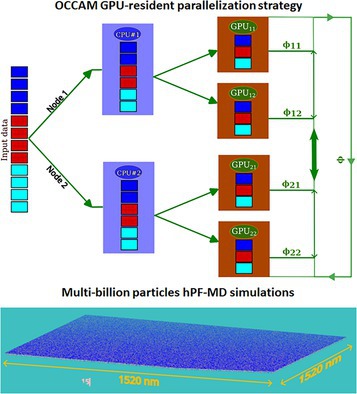

## 1 Introduction

Molecular Dynamics (MD) is a powerful tool applied in many fields. MD is mainly related, but not restricted, to investigations in chemistry, materials science, biology, and physics. The attractive feature of MD lies in its ability to describe various phenomena on the basis of underlying molecular mechanisms/structures and then give a picture of these phenomena at atomic resolution. In principle, MD could allow characterizing complex molecular behaviors and predicting macroscopic properties starting from an atomic scale model. In several cases, by acting as a link between theoretical models and actual observations, MD enabled confirming theoretical predictions with empirical evidence and vice versa. For example, MD can be an aid for the interpretation of protein data [1, 2] to improve the production of materials [3] and to develop new ones [4]. Ideally, if reliable and well‐validated force fields are available for a given class of compounds, atomistic models are the ideal way to approach this type of problem. In practice, in most cases, due to the large length and time scales related to the phenomena of interest, the use of atomistic models would require computational efforts that can be out of reach for actual computer power [5].

The range of applicability of MD or any other molecular simulation approach is determined by the computer budget and the availability of simulation codes able to exploit efficiently the hardware. The advent of multi‐core computer architectures (multi‐CPU) for common daily life applications first and, later on, Graphic Processing Units (GPU), more recently available for computing applications, triggered several efforts toward parallel computer codes for MD simulations [6, 7, 8]. Nowadays, several MD simulation software, commonly shared among the scientific research community, have stable and efficient versions able to run on parallel multi‐CPU distributed architectures, and several efforts have been made to achieve similar results on single and multiple GPU architectures [9, 10, 11, 12].

Several packages for standard MD simulations implemented GPU parallelization; the code NAMD has been one of the first and can represent the evolution of the successful strategies established during the last decade [13]. In first versions, short‐range electrostatics and Lennard‐Jones interactions were assigned to GPU acceleration, while bonded interactions and integration of the MD simulation were performed by the CPU [13]. Performance tests have shown that the CPU to GPU memory transfer is a bottleneck, which has been established as a common feature of GPU computing and is not related to a specific simulation code [6]. For this reason, subsequent versions of the NAMD code moved computation from the CPU to the GPU, achieving a so‐called GPU‐resident approach [9]. A similar approach has been implemented for GROMACS, performing the complete MD on the GPU [10, 14]. Similar paths of migration from CPU to GPU have been followed by other simulation codes, as, for example, LAMMPS [15], AMBER [16, 17] and CHARMM [18]. Differently, codes such as HOOMD [19] and ACEMD [20, 21] have been explicitly designed for the new hardware, where most of the computational work is natively performed on GPUs. The introduction of GPUs has had a significant impact also on the MD algorithms, sparking continuous efforts of the molecular simulation community to fully harness GPUs capabilities for codes previously developed for CPU architectures. The most computationally expensive part of an MD simulation is the evaluation of non‐bonded forces. Reformulating the pair interaction calculations to fully utilize GPU parallelism is a major goal for software developers. For instance, in GROMACS, significant optimizations have been introduced, such as a reformulation of the Verlet list, pair searching, and cutoff management. Specifically, the implementation of a dual pair list with rolling pruning updates has led to notable improvements in performance on both single GPUs and multi‐node applications [10]. Another important challenge is the precision required for certain applications, such as free energy calculations. For example, the primary GPU‐accelerated simulation engine in the AMBER software package (PMEMD) has been designed to ensure that the statistical and thermodynamic properties it calculates are indistinguishable from those obtained using earlier versions of the code developed for CPUs [16].

A further way to widen the application of MD is the development of coarse‐grained (CG) models. In the last two decades, several methods that are able to achieve CG models with a resolution close to an atomistic scale gave the possibility to expand the range of application of MD to the mesoscale [22]. According to the previous points, efficient parallel codes to run simulations using CG models are a very profitable combination that is able to expand the limits of MD simulations as far as possible.

**SCHEME 1:**
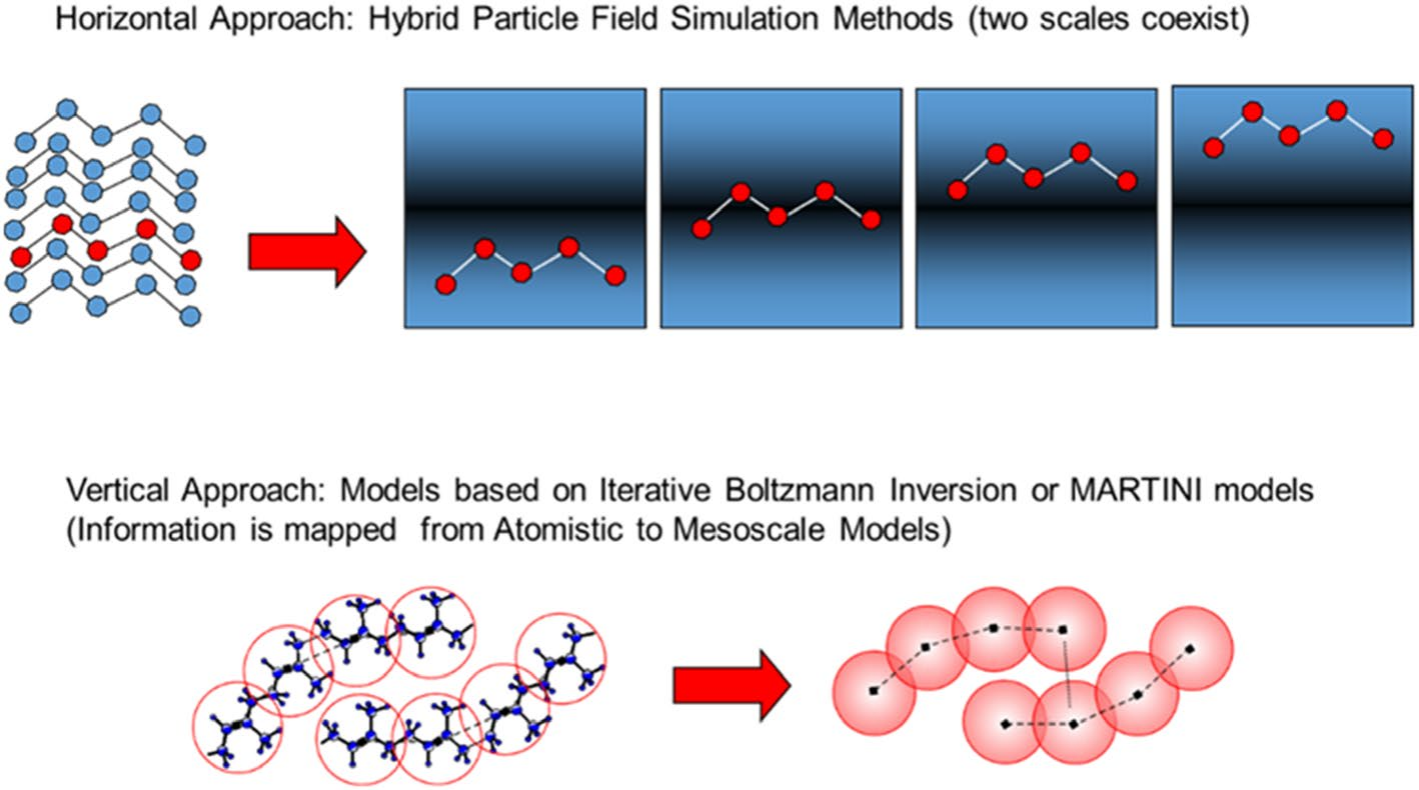
Coarse‐graining horizontal (upper panel) and vertical approaches (lower panel) are compared. In horizontal approaches, like hybrid particle‐field MD, the two scales (particle and field) coexist in the same model. In contrast, vertical approaches are based on particle reduction, and effective interactions are parametrized to reproduce properties of models on a microscopic scale.

One way to classify techniques to achieve a CG representation of the models is to define them as horizontal or vertical. A CG technique can be defined as a way to represent a molecular model in coarser representation, but able to take into account the information coming from an underlying microscopic model. In Scheme 1, the two different horizontal and vertical approaches are compared graphically. In particular, a CG approach can be defined as horizontal if the microscopic and the coarser representations coexist in the same model. Differently, the CG approach can be defined as vertical if the information is mapped, for example, from an atomistic level to a coarser model having effective particles representing several atoms. In vertical CG approaches, the computational costs of MD simulations are reduced through reduction of the number of particles. On the other hand, horizontal approaches rely on a reduction of computational cost of the evaluation of non‐bonded forces. Hybrid descriptions of molecular models combining particle and field representations are particularly suitable for horizontal CG. For this reason, if classical simulations based on pair non‐bonded interactions and a simulation based on density dependent non‐bonded interactions are compared, the latter exploits a coarser representation of the multi‐body problem. Indeed, force calculations make use of density fields that are collective variables obtained by grouping several particles (usually from 4 to 10) into one datum.

Hybrid descriptions of molecular models combining particle and field representations are particularly suitable to achieve good computational performances and to efficiently exploit parallel hardware. For this reason, Daoulas and Müller introduced the Single Chain in Mean Field (SCMF) approach combining a field representation of non‐bonded interactions routed in Self Consistent Field Theory (SCFT) and Monte Carlo simulations [23]. Later on, particle and field representations have been combined in an MD scheme named hPF‐MD [24]. Due to its hybrid formulation, hPF‐MD is particularly efficient in parallel MD simulations [25, 26]. The main reason is that the field representation used for the calculation of non‐bonded forces requires, in comparison with standard MD simulations, a low amount and less frequent data exchanges. A first parallel implementation of hPF‐MD in the OCCAM code, suitable for distributed multi‐CPU architecture using MPI, has been reported by some of us [25]. More recently, an optimized version has been documented [27]. As for GPU architecture, the first implementation of hPF‐MD has been reported by some of us for the computer code GALAMOST using CUDA C [28]. Moreover, a parallel implementation for large‐scale SCMF Monte Carlo simulations (SCMF‐MC) in the SOMA software, suitable for GPU acceleration using OpenACC, has been documented by Scheneider and Müller [29].

A large number of applications of such hybrid models have been performed for systems such as synthetic polymers, surfactants, and biomembranes [30, 31, 32, 33, 34, 35, 36, 37, 38, 39, 40]. Applications of hybrid particle‐field models include both coarse and chemically detailed representations, including also all‐atom models [41, 42, 43]. Due to their computational advantages and the large efficiency in parallel simulations, hybrid particle‐field models have been applied to problems with typical time and length scales generally not accessible to equivalent models based on pair potentials, and constant efforts are made in current literature to extend their range of applicability [27, 37, 38, 44, 45]. To have an idea about the performances in comparison with classical MD simulations based on pair potentials, parallel hPF‐MD runs on 16 and 64 CPUs, for systems of half a million beads, have execution times from 5 to 20 times faster [25]. This performance gap is larger if larger systems are simulated [27]. This work aims to document the parallelization of hPF‐MD simulations in the OCCAM code, optimizing it for execution on multi‐node, multi‐GPU architectures using CUDA Fortran. Based on recent literature results, above reported on classical MD, an efficient designing strategy is to obtain a code performing all the operations of hPF‐MD only on GPU. Moreover, in order to better exploit the GPU performances in multi‐node runs, three different layers of parallelization have been implemented: the first one among different nodes, the second one to better distribute the load on different GPUs present on different computational nodes, and the last one to exploit the intrinsic parallelism of each GPU.

The paper is structured in the following way; after the Hardware and Software Configuration section, in the first section of the discussion, entitled Hybrid Particle‐Field Molecular Dynamics, the basic theory, the main operations, and the relative algorithms needed to perform hPF‐MD are described. The second and third sections, entitled Parallelization on Different GPUs and Parallelization inside a Single GPU, describe the two different parallelization layers designed for an efficient use of the most common multi‐node, multi‐GPU hardware to perform hPF‐MD simulations. The last section of the discussion reports performance results and feasibility tests in comparison with multi‐CPU simulations, for different large‐scale systems ranging from a few million to 10 billion beads. The conclusion section highlights the main results and the future perspective applications of the parallel code here described.

## 2 Hardware and Software Configuration

For the benchmarks reported in this study, two different computing systems, provided by CINECA through ISCRA Type C projects, have been used. For multi‐million benchmarks, the Marconi 100 cluster has been used. Marconi 100 is configured with computational nodes having four NVIDIA Volta GPUs with 16 GB of memory each, hosted in a single node managed by an IBM POWER9 AC922 CPU running at 2.6 GHz. For the larger benchmarks, the Leonardo supercomputer at CINECA has been utilized. This machine allows exploiting NVIDIA A100 Tensor Core GPUs. In particular, each node of Leonardo has an Intel CPU with 32 cores (Xeon Platinum 8358) and four NVIDIA A100 Tensor Core GPUs, each with 64 GB of memory. The version of OCCAM code described in the present study has been written in CUDA Fortran and using CUDA‐aware MPI as the communication interface. Both compiler and MPI interface are included in version 24.1 of the NVIDIA HPC software development kit. All performance benchmarks have been obtained by running 50.000 MD steps and using an update frequency of density fields of 100 steps. In this way, it was possible to average timing over 50.000 force calculations (and other operations performed at each step) and over 500 density calculations and the related GPU to GPU communications needed to obtain global sums of density fields. This number of steps guarantees small deviations of the measured runtimes of about 1% between different simulation runs.

## 3 Discussion

### 3.1 Hybrid Particle Field Molecular Dynamics

This section describes the hPF‐MD technique and its main assumptions. Bonded force calculations in hPF‐MD simulations are equivalent to those employed for traditional MD simulations. This allows importing into the hPF‐MD scheme every molecular model with any complex architecture already developed for traditional MD simulations. Differently, non‐bonded forces, the most expensive part of MD simulations, are not treated using pair potentials.

The leading concept of the hPF‐MD approach is to consider a molecule interacting with neighboring molecules via a density dependent potential acting as an external field due to non‐homogeneous spatial density distributions of particles. This approach, named mean field approximation, allows a computationally convenient way to calculate non‐bonded forces in MD simulations. Indeed, double loops over particle pairs, needed to calculate forces for non‐bonded interactions, typically calculated for Lennard‐Jones potentials, in hPF‐MD simulations are substituted by single loops over particles interacting with the mean fields.

The basic ingredient of hybrid particle‐field models is the determination of the partition function of a single molecule in an external field to obtain an appropriate expression for the external potential at particle positions $V\left(r_{i}\right)$ and its spatial derivatives. For detailed derivations starting from the definition of the partition function the reader can refer to [24]. The main result of the hybrid particle‐field approach is obtained by saddle point approximation. The result of this approximation is that the density‐dependent external potential can be written as a functional derivative of total non‐bonded energy functional. In other words, if the non‐bonded energy is described as a density functional, the external potential can be calculated as a functional derivative. The main assumptions and the derivation of this result are reported in Section 3 of the Supporting Information. A common form of non‐bonded interaction, widely employed in the frame of SCFT is the Helfand functional form: [46].

(1) $$W\left[\phi_{K}\left(r\right)\right]=\int dr\left[\frac{k_{B}T}{2}\sum_{\mathrm{KK}\prime }\chi_{KK^{\prime }}\phi_{K}\left(r\right)\phi_{K\prime }\left(r\right)+\frac{1}{2\kappa }{\left(\sum_{K}\phi_{K}\left(r\right)-1\right)}^{2}\right]$$

Assuming the functional form in Equation (1) the external potential is obtained as a functional derivative in the following way:

(2) $$\begin{array}{c} {\left.\frac{\mathrm{\delta W}\left[\phi_{K}\left(r\right)\right]}{\delta \phi_{K}\left(r\right)}\right|}_{r_{i}}=\sum_{i}V\left(r_{i}\right)\\ \;=k_{B}T\sum_{i}\left(\sum_{K^{\prime }}\chi_{{\mathrm{KK}}^{\prime }}\phi_{K^{\prime }}\left(r_{i}\right)+\frac{1}{\kappa }\left(\sum_{K}\phi_{K}\left(r_{i}\right)-1\right)\right), \end{array}$$

where *ϕ_K_*, in both Equations (1) and (2), denotes the density of the species *K* normalized by the average density of the system. The Boltzmann constant and system temperature are denoted by *k*_*B*_ and *T*, respectively. Each density field, related to a given particle type *K*, is specified by the same index *K*. Similarly to the notation of Flory‐Huggins (FH) theory, $\chi_{\mathrm{KK}\prime }$ is the interaction parameter between a particle of type *K* and the density field of particles of type *K′*. The physical meaning of this parameter is similar to that in FH theory (describing the compatibility between species *K* and *K*′), but the density is a field so the interaction felt by the particle is with a local density at a position ***r***. The second addend in Equation (2), named incompressibility condition, is a penalty term introduced by Helfand. This term, where *κ* is named compressibility, penalizes differences between local density fields and average density. Easier to understand is the simple situation of a two component mixture of species *A* and *B*. In this case the external potential acting on a single particle of type *A* and located at position *r* is:

(3) $$V_{A}\left(r\right)=k_{B}T\left[\chi_{\mathrm{AA}}\phi_{A}\left(r\right)+\chi_{\mathrm{AB}}\phi_{B}\left(r\right)\right]+\frac{1}{\kappa }\left(\phi_{A}(r\right)+\phi_{B}\left(r\right)-1).$$

More specifically, for example, if the species *A* were hydrophobic particles in water (type *B*), $\chi_{\mathrm{AB}}$ (the mean field interaction parameter between a particle of type *A* and the density field of particles of type *B*) would be positive and large. The incompressibility condition produces a positive contribution to the external potential at points where the values of the normalized density $\phi \left(r\right)$ are larger than one. Conversely, spatial regions having $\phi \left(r\right)$ smaller than one will have negative contributions the external potential.

### 3.2 Implementation of hPF‐MD

The force acting on the single particle of type *A* at position *r* is:

(4) $$\begin{array}{c} F_{A}\left(r\right)=-\frac{\partial V_{A}\left(r\right)}{\partial r}=-k_{B}T\left(\chi_{\mathrm{AA}}\frac{\partial \phi_{A}\left(r\right)}{\partial r}+\chi_{\mathrm{AB}}\frac{\partial \phi_{B}\left(r\right)}{\partial r}\right)\\ \;-\frac{1}{\kappa }\left(\frac{\partial \phi_{A}\left(r\right)}{\partial r}+\frac{\partial \phi_{B}\left(r\right)}{\partial r}\right) \end{array}$$

As was explained above, the difference between traditional MD simulations and hPF‐MD lies in the evaluation of non‐bonded forces obtained by the calculation of spatial derivatives of the external potential (i.e., density fields) as outlined in Equation (4).

**FIGURE 1:**
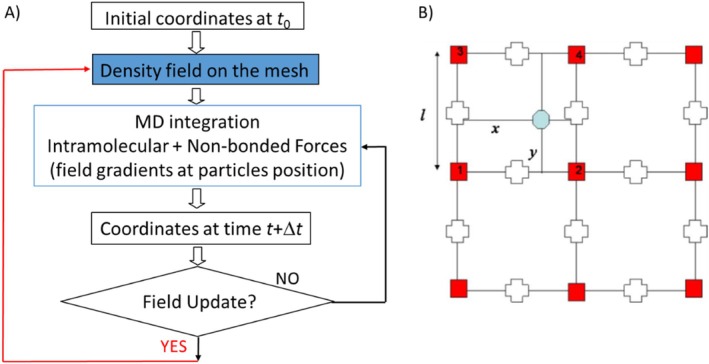
(A) Iterative scheme for hPF‐MD simulations; (B) simplified example (2D) for particle assignment to a mesh following the PIC scheme. Fraction of particles is assigned proportionally to the area of a rectangle whose diagonal is the segment connecting the particle and the lattice point on the opposite side of the cell (empty crosses indicate the staggered lattice where the derivatives are defined).

According to this, the main ingredient needed to implement non‐bonded force calculation, and then to connect particle and field models, is the obtainment of a density field from particle positions. In Figure 1A, the iterative scheme needed to perform hPF‐MD is outlined. Particle coordinates from the initial configuration (time *t*_0_) will give initial values of the density dependent external potential. The calculated forces on particles will be the sum of all intramolecular terms (bonds, angles, dihedrals, etc.) and the external potential due to the density field. From MD integration, a new configuration is obtained and a new density field, with a predetermined frequency, is obtained. This will lead to a new external potential that will be used to calculate non‐bonded forces for the next MD integration. The key part of implementing the hPF‐MD technique is the calculation of the number density values for different particle types in the space and their spatial derivatives, i.e., density fields for each species and their derivatives.

In the spirit of particle‐mesh approaches, the construction of a spatial mesh where density is described with a given resolution is a convenient procedure. In the OCCAM code, the “linked lists” approach, assuring an efficient sorting of the particles, is utilized [25]. To assure smooth behavior of the density fields, a particle in cell (PIC) approach is implemented [47], as explained in Figure 1B, assigning fractions of a particle residing in a cell to mesh points according to their position. The spatial derivatives of the density distribution are calculated on a staggered lattice, as shown in Figure 1B. Once the meshes corresponding to density fields are obtained, values of external potential and of its spatial derivatives can be interpolated at particle positions.

### 3.3 Parallelization Scheme

As for any standard MD code, the input data for hPF‐MD simulation consists of configuration and topology including force‐field parameters for bonded and non‐bonded interactions among particles. According to the hPF‐MD scheme, non‐bonded interactions are calculated using density fields. As described in the previous section, density fields for each species are defined on a three‐dimensional mesh. So further information regarding the mesh size and the mean field parameters has to be provided in the input files. It is worth noting that, as already implemented for the parallel version of OCCAM for multi‐CPU architectures [25, 27], all real variables and relative computations are defined using double precision.

A first ingredient for an efficient parallelization of the hPF‐MD simulation is a decomposition of the system coherent with both the simulation scheme and the parallel hardware employed. A proper decomposition should guarantee a most complete parallelization of the operations and, at the same time, a minimization of the amount of data communication in terms of their size and frequency. From this point of view, due to the mean field approach, the hPF‐MD technique is well suited to be effectively parallelized.

**SCHEME 2:**
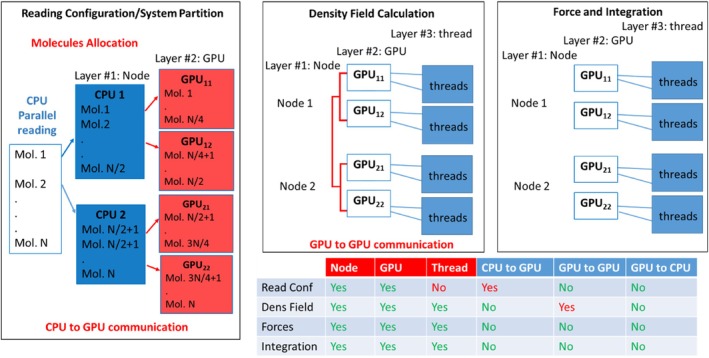
Diagrams reporting the main operations: Reading/partition (top left), density field calculation (top central), force calculation and integrations (top right) are reported. Different layers, exploited in the proposed parallelization, and communication operations needed are reported in the table on the bottom of the Scheme.

Several non‐trivial issues need to be considered to allow the large‐scale applications of hPF‐MD starting from input reading and to obtain a system partition able to fully exploit the three layers of parallelization present in multinode GPU architectures (node, GPUs on the node and threads in each single GPU). In particular, as diagrammed in Scheme 2 (left panel), the input operations have been coded in order to have a parallel reading of the configuration files. The reason is that serial reading is limited by the size of RAM of the CPU of each node. This issue was a limitation in handling system sizes larger than 100 million particles in which files have sizes of the order of terabytes.

As schematized in the top left panel of Scheme 2 (Reading Configuration/System Partition) one CPU for each node reads only the information related to the molecules assigned to the GPUs present on that node. This allows for a decrease in the RAM requirements and for handling large systems. After this operation, each GPU owns some molecules until the end of the simulation with a single CPU to GPU communication of coordinates and other molecular information operated only during the first initialization step. The bottom panel of Scheme 2 also reports a table summarizing the level of parallelization for each operation and the type of communication needed. Due to the partition obtained in the initialization step, the calculation of the density field, as schematized in the top central panel (Density Field Calculation), is fully parallelized on all three layers (node, single GPU, single thread). In particular, partial densities due to the particles owned by each GPU are calculated by a single thread for each particle. A single GPU to GPU communication step is needed (typically, according to the quasi‐instantaneous field approximation, only every 100–500 timesteps) to sum up the partial densities of each GPU. The most expensive part of hPF‐MD is the calculation of non‐bonded forces obtained by interpolating density gradients of particle positions. As schematized in the top right panel, this calculation is fully parallelized on all three layers and does not involve any type of communication step. Further details about the system partition and data structure, density field, and force calculations are reported separately in the next subsections.

### 3.4 Data Structure and System Partition

**FIGURE 2:**
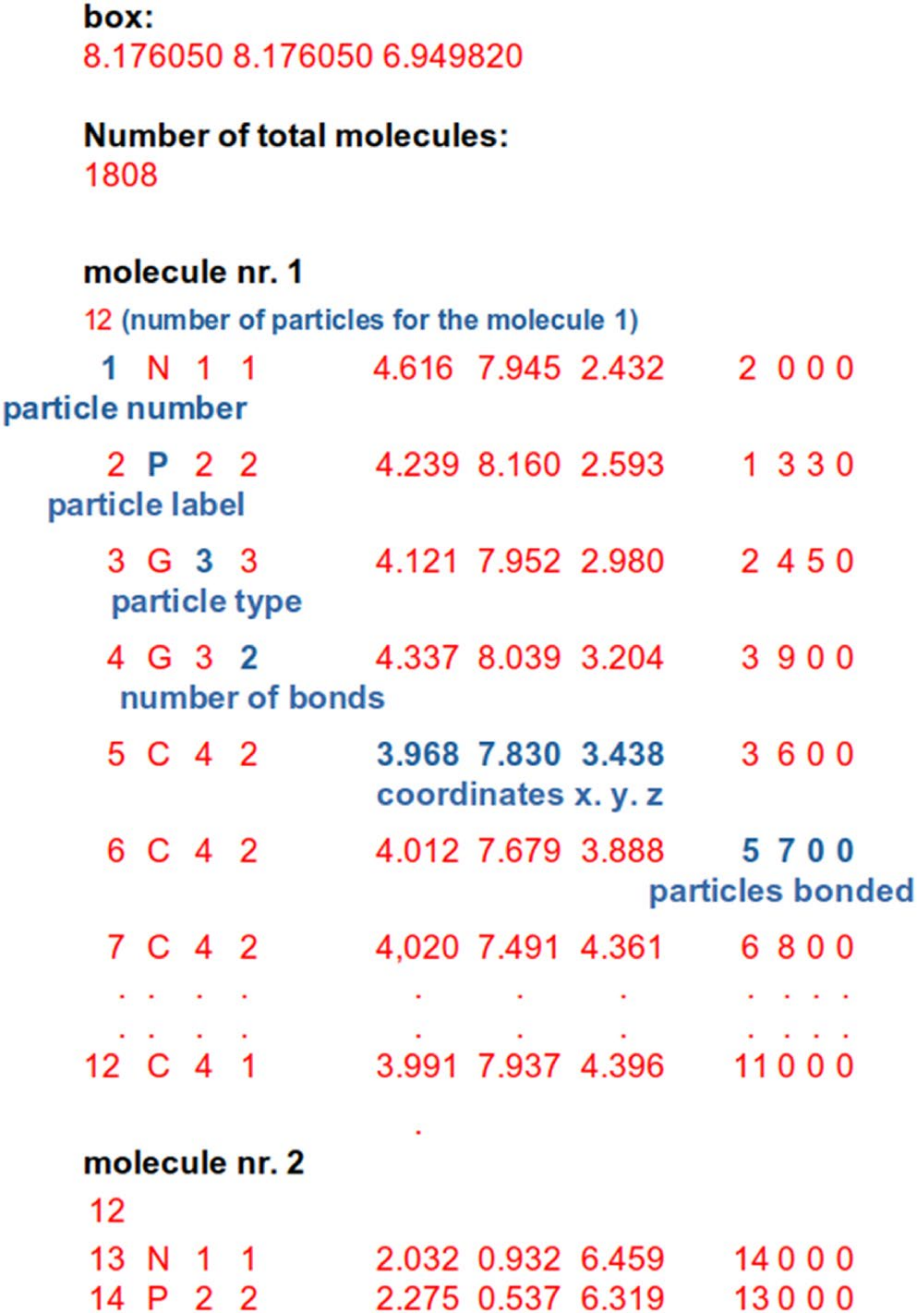
Example input configuration file for OCCAM code. On each line, all necessary information for a given particle belonging to a given molecule is provided: Particle number, label, type, number of bonds formed, Cartesian coordinates (and eventually velocities) and connectivity (index of the particles forming bonds with the particle).

The configuration file of the OCCAM code contains the coordinates of atoms collected separately for each molecule together with atom type, number of bonds formed, and their connectivity. A schematic view of this file is shown in Figure 2.

**FIGURE 3:**
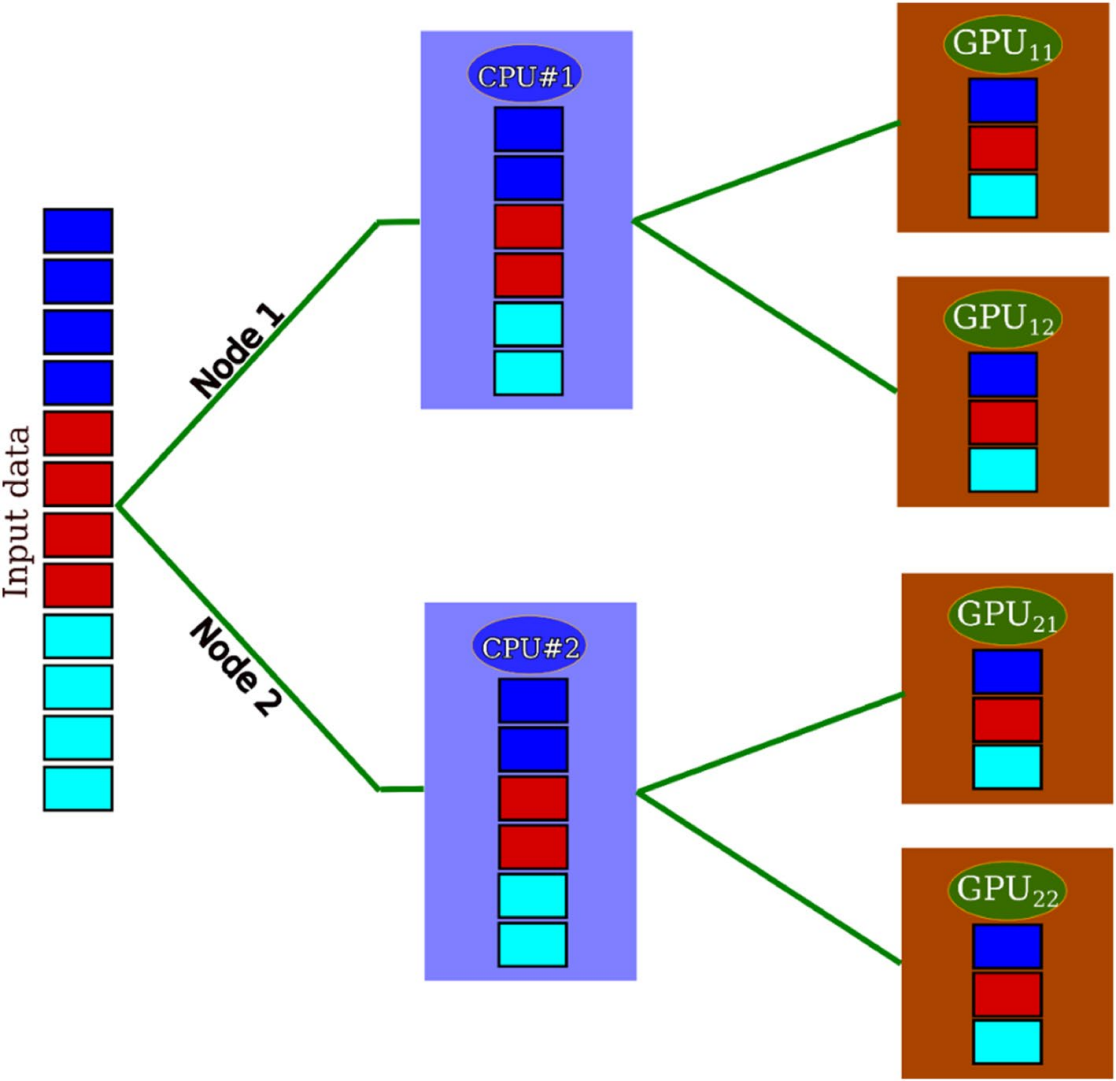
Input data describing the topology, position, velocity, and thermodynamic ensemble of the simulation molecules distributed among the nodes. In each node, a single CPU is responsible for partitioning the partial data among the GPUs.

Using the information contained in the configuration file, the code can generate, according to atom types, their connectivity and interaction types defined in the model file, a list of bonds (*bond_list*) consisting of indexes of atom pairs for each bond interaction. If angle potentials are defined, a list of angles (*ang_list*) consisting of indexes of three atoms for each angle interaction is generated. Similarly, if dihedral potentials are defined, a dihedral (*dih_list*) angle list is generated. To prepare for parallel execution as depicted in Figure 3, a single CPU from each node extracts data related to a subset of molecules and generates the corresponding *bond_list*, *ang_list*, and *dih_list*. The data generated on a given node are then further split, copying portions of them on the GPUs owned by the node. At the end of these operations, coordinates and the lists of bonded interactions of all the molecules of the system are equally distributed among the nodes assigned to the parallel job and reside only on the GPUs. It is worth noting that information about a given molecule and the corresponding particles are assigned only to a given GPU and are not split or shared. This molecule decomposition scheme is ideal for parallel jobs using hPF‐MD methodology. This point and its relevance will be clearer later.

The system partition previously described and the related input operations, as previously discussed, allow not only handling very large configuration files (of the order of terabyte), but the molecular decomposition scheme can efficiently parallelize the computation of the density fields. For traditional MD simulations, the parallelization is aimed at redesigning the pair interaction calculation in a way to provide algorithms with load balancing capabilities and a low amount of communication. In the present case, for hPF‐MD simulations, particle coordinates are never shared between nodes or between GPUs on the same node. In fact, only density fields need to be shared to calculate non‐bonded forces on particles. For this reason, differently from traditional MD simulations, domain decomposition schemes would not be useful in hPF‐MD and would not fully exploit the advantages of this technique. Sharing parts of molecules on different GPUs or different nodes would imply frequent communications of data (particle positions at each timestep) for the calculation of bonded terms, causing a large decrease in performances.

### 3.5 Parallelization of Bonded and Non‐Bonded Forces on a Single GPU

**FIGURE 4:**
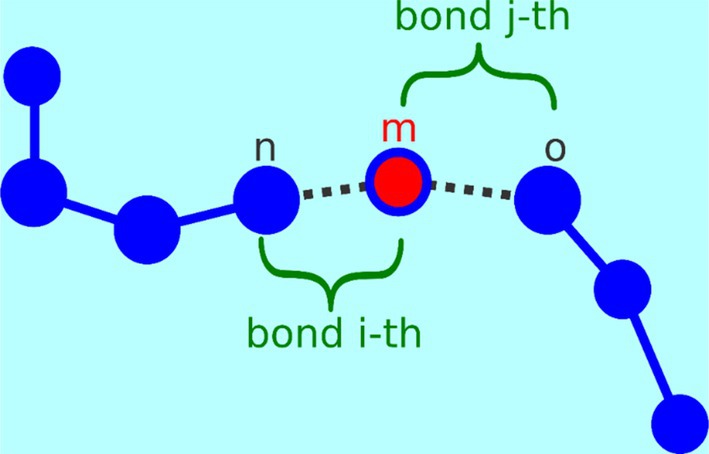
Dashed lines represent bonds between particles from two different elements in the bonding list sharing one atom. Each bond is assigned to a separate thread for concurrent computation. The threads calculate contributions to the bonding force acting upon the atom “*m*”, highlighting the need for atomic functions to maintain data consistency during parallel processing.

According to the partition scheme explained above after the first parallelization layer between different nodes, the second layer is obtained because each GPU on each node owns some molecules and can process the calculation of bonded forces components on each particle belonging to a given molecule. Finally, a third layer of parallelization is due to the GPU intrinsic parallelism. Indeed, bonded force calculations are executed via a CUDA kernel function. In CUDA programming, when a kernel is initiated, multiple threads are generated, following a predefined grid and block configuration. These threads execute the same function concurrently. Particularly, each thread is responsible for one element from the bonding list, specifying the atoms involved in bonds for which it computes the corresponding bonding forces. In Figure 4, “bond *i*‐th” and “bond *j*‐th” denote two distinct bonds sharing one atom (*m*) in the middle. Each of the two atom pairs is assigned to a different thread. However, both threads contribute to computing the bonding force acting upon the atom “*m*”. Therefore, they may attempt simultaneous access and modification of the same array element corresponding to the bonding force acting on atom “*m*”, potentially causing data consistency issues. To tackle this challenge, the use of the atomic function “atomicadd” is employed. This function reads and writes the value of its operand in device global memory.

During this single atomic operation, a thread temporarily blocks access for the other threads to the shared array element and performs the necessary operations on it. This ensures that only one thread at a time can modify shared data, thus eliminating the risk of conflicts or data inconsistencies. A similar situation is obtained for angle and dihedral forces; also, in this case, the use of atomic functions is employed.

**SCHEME 3:**
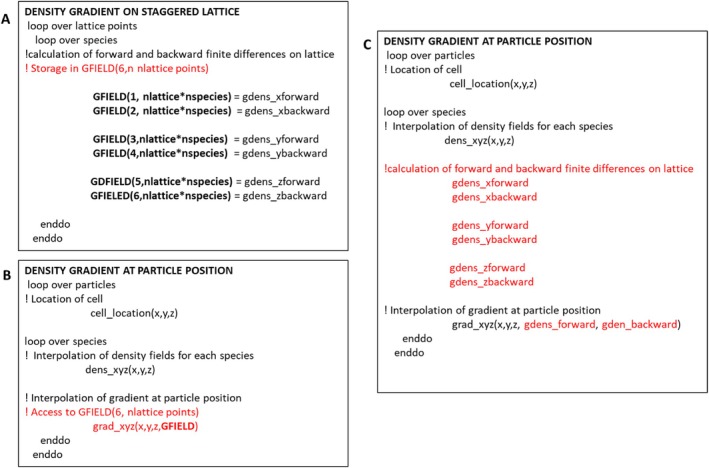
Pseudocodes describing the previous implementation for the calculation of: (A) density gradient, (B) interpolation of density and gradient at particle position compared with (C) proposed memory saving implementation.

The determination of non‐bonded forces relies on the computation of the gradient of the density field at the particle's position through linear interpolation. Non‐bonded forces acting on particles are evaluated by interpolating the spatial derivatives of the density field for each species at the particle position. Preliminary tests have shown good performance of the multi‐GPU implementation of non‐bonded force calculation as previously implemented in the multi‐CPU version of OCCAM and GALAMOST. The main problem of the previous implementations is a bottleneck to perform multi‐billion simulations due to memory occupation. The main source of memory occupation is the storage of large vectors of real numbers related to density fields when they are calculated for large box sizes. It is worth reminding that real numbers and all the relative calculations in the OCCAM code are executed using double precision. This allowed the use of hybrid particle‐field models for thermodynamic integration to study phenomena usually inaccessible to traditional MD simulations, such as self‐assembly of nanoparticles in polymer melts [48, 49] and nanoplastics aggregation in water [50]. To better explain the proposed implementation, in Scheme 3, pseudo‐codes showing the approach used in the previous OCCAM multi‐CPU and the proposed GPU implementation are compared.

According to the previous implementation, at each update after the calculation of the density fields, the forward and backward finite differences along the three different Cartesian directions are stored in a matrix (GFIELD in the panel A of Scheme 3 having size [6, nlattice*nspecies]). As shown in the pseudocode of panel B of Scheme 3, the interpolation of the density gradient at the particle position needed to evaluate the non‐bonded forces is obtained in two steps. First, using the particle coordinates, the cell index where the particle is located is obtained. According to the cell index, the matrix GFIELD is accessed, and the interpolation of the gradient is calculated at the particle position using the six values of forward and backward derivatives stored in the matrix. In order to allow simulations of large systems, the new implementation does not involve any memory storage, so the matrix GFIELD is not allocated. In particular, as reported in the pseudocode of panel C of Scheme 3, the six values of the forward and backward derivatives are calculated before the gradient interpolation and after the identification of the cell index. This implementation exploits the high parallelism of GPUs (each thread execute the calculations for each particle) without weighing down the memory when the system is large. A CUDA kernel function is dedicated to the calculation for the particles owned by each GPU of forces due to the density field. This process unfolds in two steps: in the first step, each particle is assigned to a thread responsible for identifying its grid cell within the grid designated for density field calculations and its precise position within that cell. Subsequently, these pieces of information are utilized to compute the gradient of the density field at the particle's position through linear interpolation. Finally, the forces acting on each particle processed by each GPU are computed in relation to the field gradient density. Importantly, each of these calculations operates independently of the others. Also in this case, according to the partition scheme, there are different levels of parallelization; the first one is related to parallel force calculations of different molecules allocated on different GPUs (on the same or on different nodes). The second type is due to the GPU intrinsic parallelism.

After the calculation of bonded and non‐bonded forces, each GPU independently, for the molecules allocated, employs a CUDA kernel function to compute a new particle configuration.

We would like to stress that, for all the operations described, the calculations are fully parallelized on all layers (for each node, for each GPU in the same node and for each thread inside GPUs) and, according to the hPF‐MD scheme, do not involve any communication. Indeed, due to the molecular decomposition scheme, only a whole molecule (not a part of it) is completely allocated on one GPU. This choice implies no communication among different nodes and among different GPUs of the same node as well, of particle positions for the calculation of bonded terms. In this framework, following the proposed parallelization strategy, the only communication operation needed is the update of density fields corresponding to the newly acquired configuration. The implementation of this component requires careful attention due to its substantial impact on performance and will be further discussed in the subsequent subsection.

### 3.6 Density Evaluation From Partial to Global

As explained above, in hPF‐MD simulations, non‐bonded potentials depend only on density fields, which are defined on a three‐dimensional mesh. Density values on the mesh points are evaluated by counting particles in given cell and assigning fraction of them according to their position. According to the proposed parallelization scheme, on each *j*‐th GPU on the *i*‐th node (GPU_*ij*_) the evaluation of the densities on the mesh points is executed only for the molecule assigned to that GPU. These values are defined as partial density fields $\phi_{\mathrm{ij}}\left(r\right)$ where ***r*** is the position of the lattice point and the indexes *i* and *j* refer to the molecules assigned to the GPU_*ij*_. In this way, the density corresponds only to the particles owned by that GPU. A CUDA kernel function is employed to compute $\phi_{\mathrm{ij}}\left(r\right)$ and each thread handles one particle.

**FIGURE 5:**
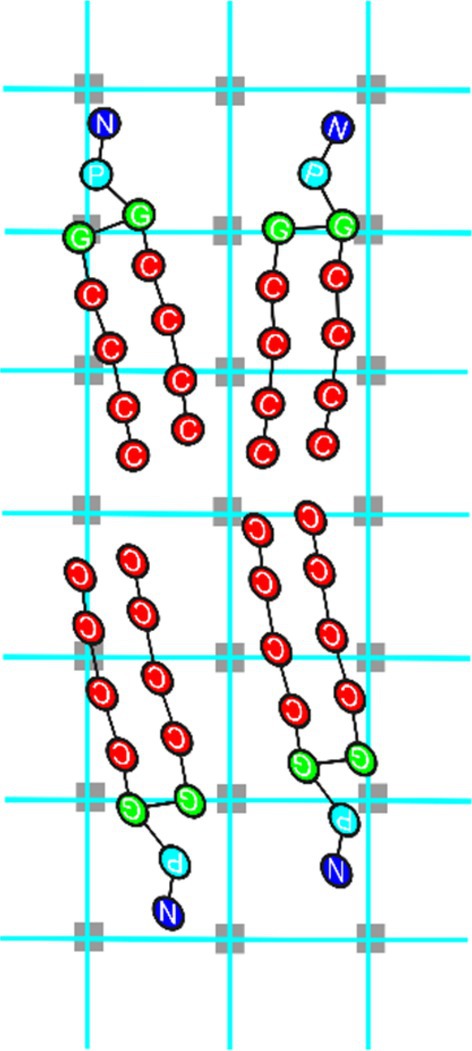
Illustration of a possible spatial distribution of some molecules assigned to a GPU and corresponding lattice points (in transparent gray) where the density is assigned. Usually, more than one particle contribute to the density field calculated at the same lattice point.

Figure 5 displays molecules processed by the GPU_*ij*_. Usually, in current application of hPF‐MD, where usually each cell contains from four to 10 particles, it happens that more than one particle can contribute to $\phi_{\mathrm{ij}}\left(r\right)$ at the same lattice points, resulting in multiple CUDA threads attempting simultaneous access and modification of the same array element. Also for this case, atomic functions are employed to avoid potential concurrent data access and modification by different threads.

**FIGURE 6:**
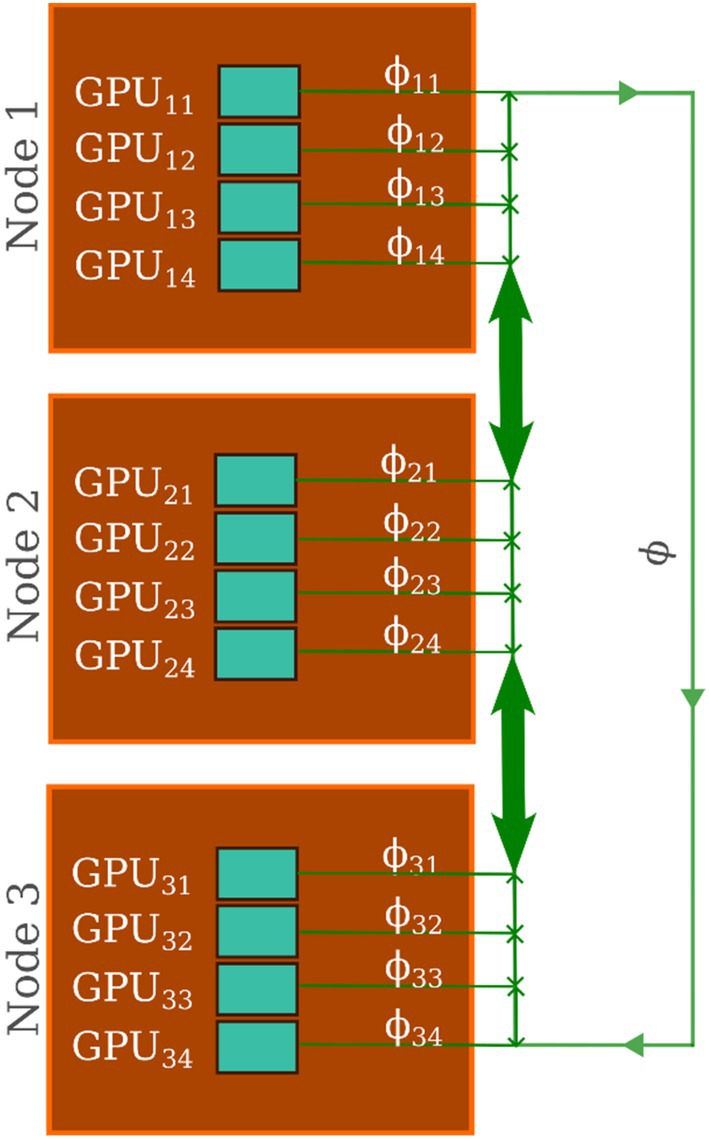
Multi‐node, multi‐GPU parallelization scheme adopted for the calculation of the density fields. The partial density $\phi_{\mathrm{ij}}$ calculated from molecules owned by GPU *j* of node *i* (GPU_*ij*_). The total density field ϕ, calculated from all molecules in the simulated system, is obtained by summing up partial densities $\phi_{\mathrm{ij}}$ using the CUDA‐aware technology, which enables efficient data exchange among GPUs within the same node and among GPUs belonging to different nodes.

Once that each GPU completed the calculation of the partial density field $\phi_{\mathrm{ij}}\left(r\right)$, in order to have global density field, a summation $\phi \left(r\right)=\sum_{\mathrm{ij}}\phi_{\mathrm{ij}}\left(r\right)$ of partial densities is needed. This summation involves the variables $\phi_{\mathrm{ij}}\left(r\right)$ residing on different GPU memories of different nodes. It is important to stress that, according to the proposed scheme, particle positions and velocities are not part of any communication step. Only partial densities $\phi_{\mathrm{ij}}\left(r\right)$ fields (objects much coarser than positions and velocities) have to be summed for the calculation of non‐bonded interactions. Moreover, to achieve computational efficiency for the summation operation, CUDA‐aware MPI is employed, as illustrated in Figure 6.

This technology integrates MPI communication with CUDA kernels, optimizing data transfer and synchronization between GPUs across the computing cluster. Consequently, CUDA‐aware MPI significantly accelerates the performance of the molecular dynamics code. A key benefit of CUDA‐aware MPI is direct GPU‐to‐GPU communication, bypassing the slower host memory. This is especially advantageous for large‐scale molecular dynamics simulations due to the large data volumes involved. By minimizing data exchange between GPUs and the host, CUDA‐aware MPI substantially reduces communication overhead and improves overall application performance.

Another computational advantage, related to the collective nature of the density fields, is that partial density calculations and their sum are not performed at each timestep of the MD simulation. The motion of single particles is faster than the evolution of the density fields. This feature leads to the “*quasi instantaneous field approximation*” [23], for which, without loss of accuracy, it is possible to define a frequency for the update of the density field. For typical applications, this frequency is between 100 and 500 timesteps. This furtherly reduces the total amount of data exchange during the simulations.

## 4 Results

To assess the performances for the proposed implementation, several benchmark simulations of lipid and water mixtures have been carried out. Details about these systems are reported in Tables 1, 2, 3. A description of the models employed for the benchmarks is reported in Section 1 of the Supporting Information material.

**TABLE 1:** Computational performances of reduced and atomic sums for calculations of global physical properties.

			Composition			
No. of particles ×10^6^	Cubic box length (nm)	No. of lattice points	No. of lipids	No. of water ×10^5^	Weight % lipids	Computing time ratio^a^
1	48	82^3^ = 551.368	18.750	7.75	29.0	0.65
2	61	103^3^ = 1.092.727	37.500	15.5	29.0	0.63
5	82	140^3^ = 2.744.000	93.750	38.75	29.0	0.63
7	9	157^3^ = 3.869.893	131.250	54.25	29.0	0.63
10	104	177^3^ = 5.545.233	187.500	77.50	29.0	0.60
20	131	223^3^ = 11.089.567	375.000	155.0	29.0	0.59

^a^ Ratio between time for reduced sum over time for atomic sum.

**TABLE 2:** System parameters for the coarse‐grained lipid‐water model used to evaluate OCCAM performances.

			Composition			
No. of particles ×10^8^	Box size (nm^3^)	No. of lattice points	No. of lipids ×10^6^	No. of water ×10^6^	Weight % lipids	System
0.6468	379 × 379 × 50	403 × 403 × 53	0.461	59.14	0.78	LB1
1.391	556 × 556 × 50	592 × 592 × 53	0.991	127.2	0.78	LB2
2.762	784 × 784 × 50	834 × 834 × 53	1.968	252.6	0.78	LB3
5.565	1112 × 1112 × 50	1183 × 1183 × 53	3.965	508.9	0.78	LB4
10.35	1520 × 1520 × 50	1612 × 1612 × 53	7.373	946.3	0.78	LB5

**TABLE 3:** Description of systems modeling coarse‐grained water used to evaluate OCCAM's performances.

No. of particles ×10^8^	Box sizes (nm^3^)	No. of lattice points
1.56	259 × 259 × 259	221^3^ = 10.764.018
3.12	327 × 327 × 327	278^3^ = 21.513.119
6.24	412 × 412 × 412	350^3^ = 42.996.425
1.25	519 × 519 × 519	441^3^ = 85.933.265
25.0	653 × 653 × 653	556^3^ = 171.747.443
50	823 × 823 × 823	700^3^ = 343.256.876
70	920 × 920 × 920	783^3^ = 480.397.958
80	962 × 962 × 962	819^3^ = 548.952.930
100	1036 × 1036 × 1036	882^3^ = 686.038.061

### 4.1 Performance Comparison: Reduction Summation vs. Atomic Functions

The computation of global physical properties within the system, such as temperature, kinetic energy, potential energies associated with both bonded and non‐bonded interactions, necessitates data exchange stored in the memory of different GPUs, even those distributed across separate nodes. In this scenario, two distinct approaches have been employed for intra‐GPU data exchange: one relies on the utilization of atomic functions, while the other harnesses the technique of reduction summation for parallel computation [51, 52]. Reduction summation, a widely adopted technique, efficiently performs associative and commutative operations on elements within arrays across multiple threads and blocks. This method is particularly valuable in scenarios demanding the aggregation of substantial data volumes into a singular result, a common necessity in molecular simulations. Subsequently, the partially processed data from various GPUs and nodes converge into a single value utilizing CUDA‐aware technology [53].

**FIGURE 7:**
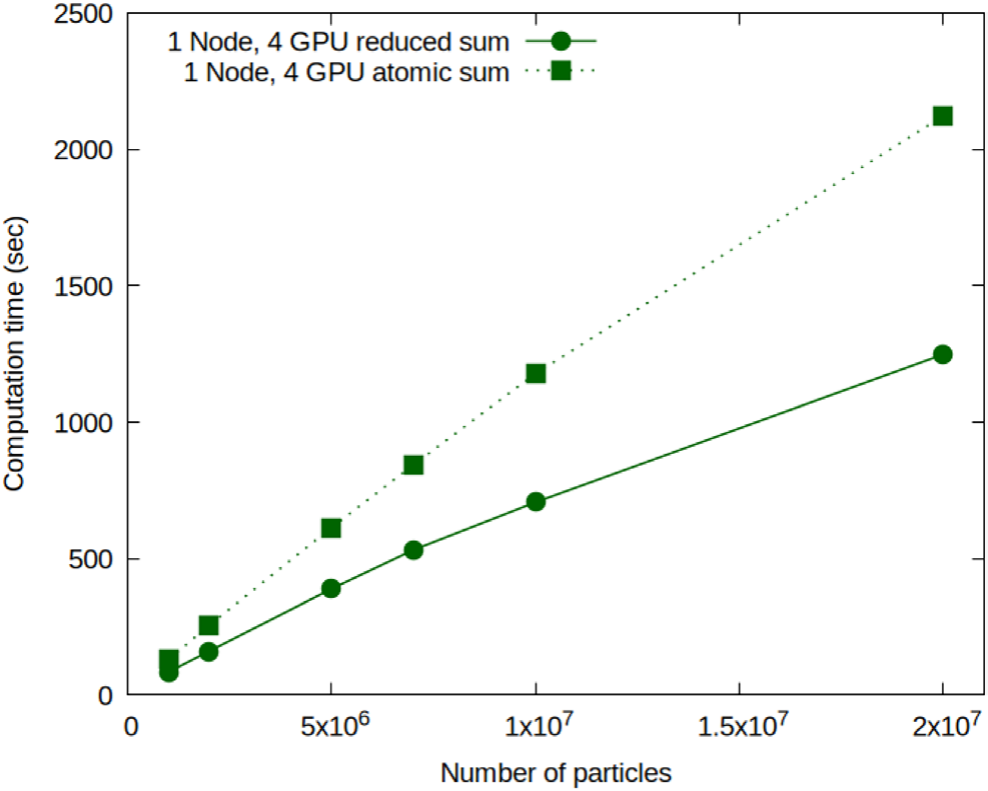
Performances comparison between reduced and atomic sums for calculations of global physical properties in a molecular simulation system.

Figure 7 compares the computational performances of each of these two approaches. Specifically, it depicts the variation in computation time as a function of the number of particles in the system, which consists of a realistic composition of surfactants in a water solution. The number of particles in the investigated systems ranges from 10^6^ to 2 × 10^7^, while the number of grid points, where density fields are defined, ranges from 82^3^ = 551.368 to 223^3^ = 11.089.567. More details regarding the number of particles, composition of lipid‐water system, simulation box size, and the number of lattice points are provided in Table 1. The computation times in Figure 7 correspond to 5 × 10^4^ MD steps performed in the NVT thermodynamic ensemble, with density updates based on particle positions occurring every 100 time steps. The computing system comprises four NVIDIA Volta GPUs with 16 GB of memory each as configured on the Marconi100 cluster at CINECA [54]. More information about the hardware is reported in the dedicated subsection entitled Hardware and Software Configuration. The solid green curve represents the results obtained using the reduction summation technique, while the dotted green curve presents the results obtained using atomic functions to compute the global properties of the simulated particle system. The reduction summation approach consistently yields lower computation times compared to the atomic function approach. Specifically, when comparing the two computation times, as shown in Table 1, it is clear that the reduction summation approach achieves an average computation time that is 63% of the time required with atomic functions. Validations of the accuracy comparing numerical results (sum of bond energies and sum of angle energies over all molecules along a MD simulation) obtained using atomic function and reduction summation are reported in Section 2 of Supporting Information material (Figures S2 and S3). In particular, numerical results for both cases are equal within the accuracy of double precision representation.

### 4.2 Multi‐GPU Multi‐Node Performances: Multi‐Million Systems

To effectively evaluate the performance of the multi‐node multi‐GPU OCCAM code, it is crucial to compare it with a CPU‐based reference model. The selected reference model is the multi‐CPU version of OCCAM [25, 27] executed on the Marconi100 cluster (see subsection Hardware and Software Configuration for more information). The systems used for this study are identical to those used to obtain the data presented and previously analyzed in Figure 7. Correspondingly, the simulation box and the density field grid sizes are those illustrated in Table 1.

**FIGURE 8:**
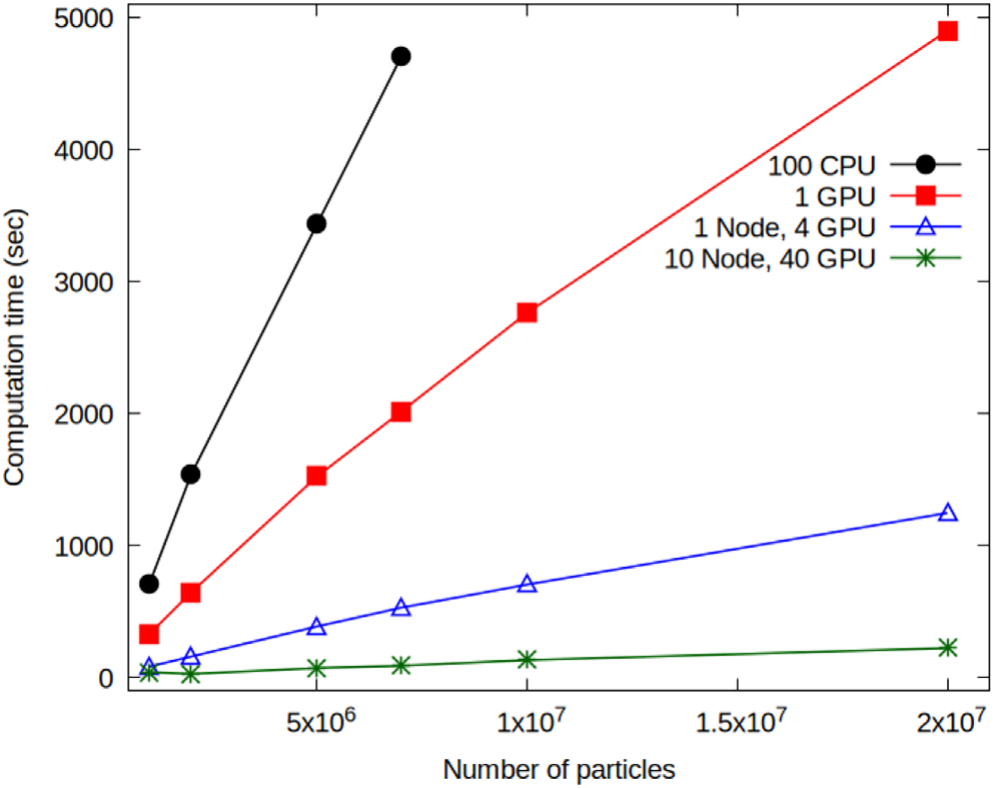
Computation times as a function of particle number: Performances of multi‐CPU OCCAM on 100 cores (black); results obtained from different multi‐GPU setups: One GPU (red), 4 GPUs one node (blue), 40 GPUs 10 nodes (green.)

Figure 8 depicts the computation times as a function of particle number obtained using the OCCAM multi‐CPU code and 100 cores (black curve). Results from different multi‐GPU setups are also presented. Specifically, the red curve represents results from a single GPU, the blue curve illustrates the trend from four GPUs on a single node, and the green curve shows results using 10 nodes, totaling 40 GPUs. Notably, the OCCAM multi‐CPU data is limited to 7 × 10^6^ particles due to the account restrictions on available RAM for a single user on the Marconi100 cluster. Conversely, results for different GPU configurations explore a maximum of 2 × 10^7^ particles. This limit is imposed by both the available memory per individual GPU and the necessity to define all arrays required for calculating the mean field potential for each particle type of the investigated system. The data presented in Figure 8 demonstrates a larger reduction in computation time as the number of particles increases when GPUs are employed. Specifically, the computation times of 5 × 10^4^ MD steps for 1 million particles are 706.3 s for 100 CPUs, 329 s for 1 GPU, 83.79 s for 1 node with 4 GPUs, and 41.88 s for 10 nodes with 40 GPUs. This trend continues as the particle number increases to 2 million, 5 million, and 7 million. It is notable that utilizing a higher number of GPUs substantially reduces the computation time compared to both CPU‐only and single‐GPU configurations, demonstrating the scalability and efficiency of parallel computing, especially when employing multiple GPUs across nodes. Namely, for systems up to 7 million particles, where execution times are available also for 100 CPU runs, a reduction of the execution time of ~2.5 for runs with a single GPU, ~10 on a single node (4 GPUs) and from 20 to 50 on 10 nodes (40 GPUs) is obtained.

### 4.3 Multi‐GPU Multi‐Node Performances: Multi‐Billion Systems

In this sub‐section, benchmarks on large‐scale systems reaching multi‐billion particles are reported. The aim is to show the reliability, using the proposed parallelization strategy, of MD simulations of considerable size. For these systems, the Leonardo supercomputer at CINECA [55] has been utilized. This machine uses NVIDIA A100 Tensor Core GPUs, each with 64 GB of memory (more information about Leonardo is reported in Hardware and Software Configuration subsection). The memory size of the GPU allows handling larger systems and transferring data than the V100 GPUs.

**FIGURE 9:**
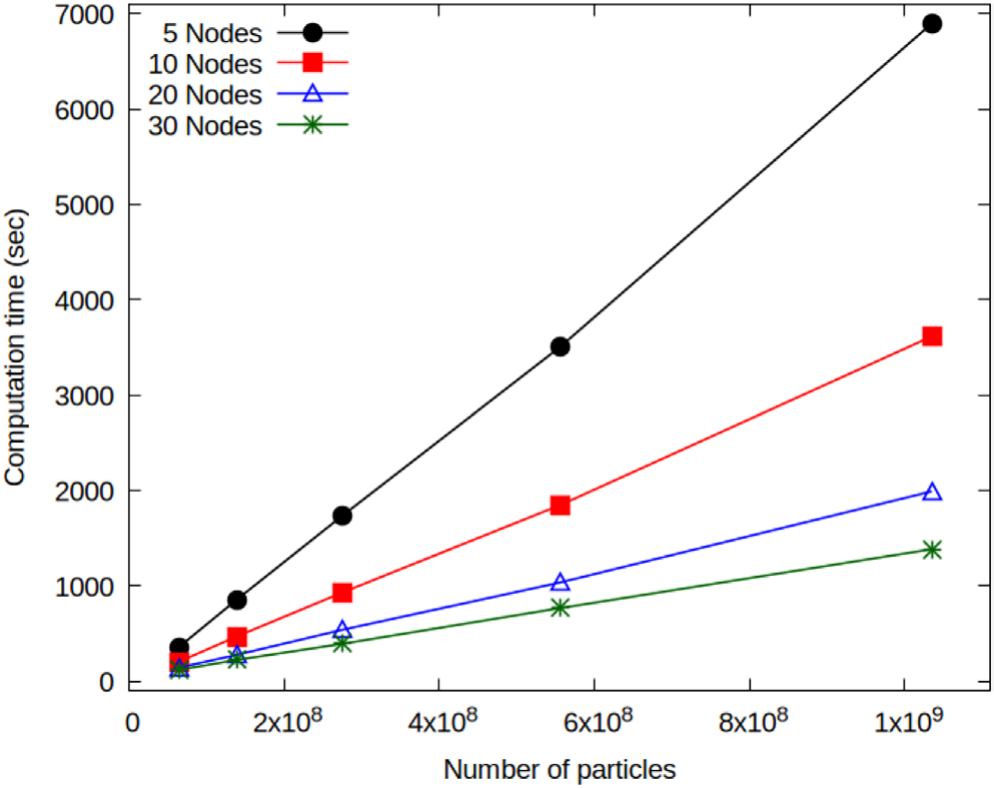
Computation times (5 × 10^4^ timesteps) vs. total Particle for different number of multi GPU nodes (each node 4 GPUs): 5 (black), 10 (red), 20 (blue) and 30 nodes (green) have been employed.

To evaluate the performance of the OCCAM code on this enhanced hardware, a CG model representing phospholipids and water has been employed. The lipid‐water system composition encompasses five distinct particle types and has been configured within the bilayer phase stability range. Performance assessment of OCCAM involves scaling up the total number of particles in the system until reaching the memory capacity limit of the GPUs. Table 2 provides a comprehensive summary of the total particle number, including lipids and water, along with details on simulation box dimensions and lattice point quantities. Figure 9 reports the computation times for 5 × 10^4^ MD steps as a function of the total particle number across varying numbers of nodes, with each node equipped with four GPUs.

**FIGURE 10:**
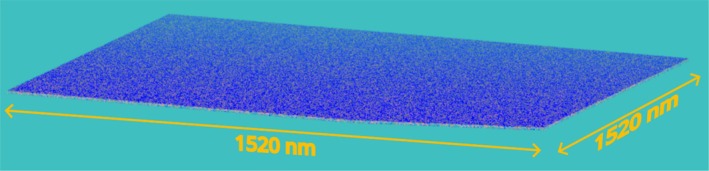
Snapshot of a coarse‐grained model of a DPPC lipid bilayer in water. The number of beads of the depicted system is 1.03 × 10^9^ and the benchmark simulation has been run for 1 million timesteps.

Specifically, the black and red curves denote the results obtained using 5 and 10 nodes, respectively; the blue curve reports trends for 20 nodes, and the green curve for 30 nodes performing MD in the NVT thermodynamic ensemble, with density updates based on particle positions occurring every 100 steps. A quantitative analysis reveals that the slopes of the linear curves decrease by approximately a factor of 1.92 going from five to 10 nodes, 1.84 going from 10 to 20 nodes, and 1.46 going from 20 to 30 nodes. These values are, of course, always smaller than the maximum theoretical value of 2 for the first two cases and 1.5 for the last case, but still very close to it. The increased number of particles in classical MD simulations largely heightens the data transfer time between the different GPUs across different nodes. On the contrary, for the present case, the nature of the hPF‐MD method together with the proposed implementation are both able to alleviate this drawback. In particular, the amount of data to be communicated for the calculation of non‐bonded forces are only density fields corresponding to several particles assigned to a given GPU and not particle positions; moreover, this communication is not operated at every timestep. Finally, the communication operations among different nodes are speeded, in the proposed implementation, by the application of the CUDA‐aware technology [53]. According to the results shown in Figure 9, a further noteworthy outcome is undoubtedly the possibility to run a system with a particle number larger than 1 billion by using only 5 computing nodes and 20 GPUs. In each explored node configuration, the particle number reaches 1.03 × 10^9^, with the computed number of MD steps per second of 7, 14, 25, and 36 for the 5, 10, 20, and 30 nodes, respectively. The last two sets of benchmarks presented in this sub‐section are based on a CG representation of DPPC lipid bilayers and a monoatomic fluid at an equilibrium density of the CG water model (both systems mapping are based on MARTINI model) of different sizes. The four‐to‐one mapping scheme adopted for MARTINI (each CG beads corresponds to four heavy atoms) allows keeping detailed chemical features in the CG representation. Several studies obtained using such mapping in combination with field representation of non‐bonded interactions have shown that this strategy is able to characterize mesoscale phenomena and explain them based on detailed molecular mechanisms. Lipid bilayers are the most common models used for molecular simulations to represent biomembranes. Using this type of CG models of lipid bilayers, due to the computational efficiency of field representation of non‐bonded forces, several large‐scale phenomena such as the inclusion of carbon nanotubes inside cells, biomembrane solubilization induced by surfactants, and drug delivery have been studied [26]. More in general, self‐assembly of both synthetic or biological surfactants, of nanoparticles and the corresponding nanoscale structures have been characterized at a detailed molecular level [48, 56, 57]. Typical system sizes employed in these studies range from 200 thousand to 8 million beads and box sizes up to the order of ~100 nm, typically running on a number of CPUs going from 48 to 864 [27]. In principle, due to the good scaling of the parallel implementation of hPF‐MD, larger systems on the scale of 100 million to 1 billion particles could be afforded by employing a larger number of CPUs on the order of thousand‐hundred thousand. From a practical point of view, for the real case of systematic studies (not simply code benchmarks), where several long simulations are needed, this amount of resource request and its use is usually quite limited. According to this point, to gain a practical understanding of the range of applicability of the multi‐node implementation here proposed, a bilayer system having 1.03 billion particles has been benchmarked using a moderate number of nodes going from 5 to 30. This system corresponds to a very large biomembrane patch of 1.5 × 1.5 μm (a snapshot of this system is depicted in Figure 10, where details about system composition are reported in the last row of Table 2).

For this system, running 1 million time steps of a simulation takes 38.3 h on 5 nodes, 20.1 h on 10 nodes, 11.1 h on 20 nodes, and 7.7 h on 30 nodes (this corresponds, considering the typical time step of 0.03 ps employed for hPF‐MD of these systems, to a trajectory of 93 ns/24 h).

**FIGURE 11:**
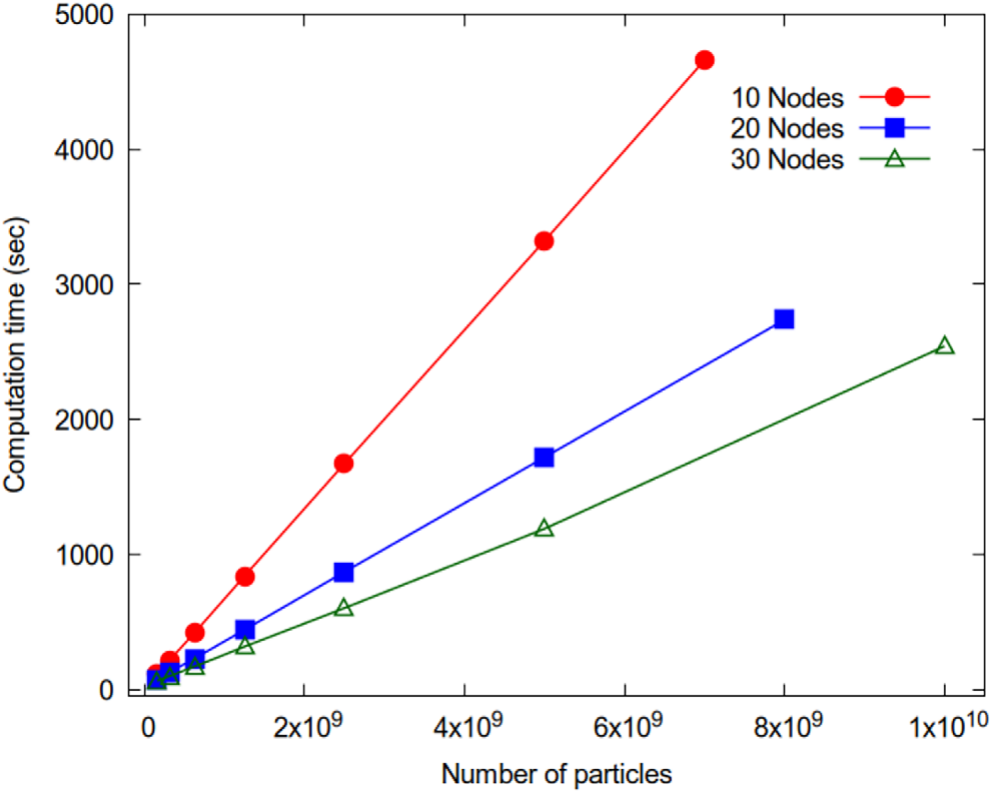
Run times (50.000 steps in the NVT ensemble) for CG water model as function of number of particles for 10, 20, and 30 nodes (red, blue, and green curves, respectively).

Simulations of the CG water model range from 156 million to 10 billion beads, having cubic box lengths going from 259 nm to 1.036 μm. Table 3 summarizes details about these systems. The runtimes for 50.000 MD steps conducted in the NVT thermodynamic ensemble are reported in Figure 11.

The red curve corresponds to results obtained using 10 nodes, the blue curve depicts outcomes from 20 nodes, and the green curve results from 30 nodes. The maximum particle number that can be handled depends on memory occupation needed to store the density fields and position/velocities (real double precision variables) of the particles assigned to a given GPU. According to the implemented parallelization scheme, more nodes are employed, and smaller is the memory occupation related to position/velocities. In this way, the maximum size of the system is related to the number of computing nodes employed. In the case of 10 nodes and 40 GPUs, the limit of 7 billion particles is achieved. This limit increases to 8 billion particles for 20 nodes and 80 GPUs, and it reaches 10 billion particles for 30 nodes and 120 GPUs. The computation times obtained are 10.7, 18.3, and 19.7 MD steps per second for 10 nodes and 7 billion particles, 20 nodes and 8 billion particles, and 30 nodes and 10 billion particles, respectively. Based on these results, running 1 million time steps of simulation takes about 25.9 h for 7 billion particles on 10 nodes, 15.2 h for 8 billion particles on 20 nodes, and 14.1 h for 10 billion particles on 30 nodes (this would correspond to a trajectory of 50 ns/24 h). For the sake of comparison, we can consider typical computational set‐ups recently employed in traditional MD simulations. To obtain similar performances, for systems of comparable sizes, simulations of 305 million particles have been run on 4096 nodes equipped with V100 GPU accelerators of the SUMMIT machine of Oak Ridge Lab [58]. As for multi‐CPU parallel applications, MD simulations of 1 billion atom systems have been run on 130.000 processor cores at the Oak‐Forest PACS supercomputer center [59].

**FIGURE 12:**
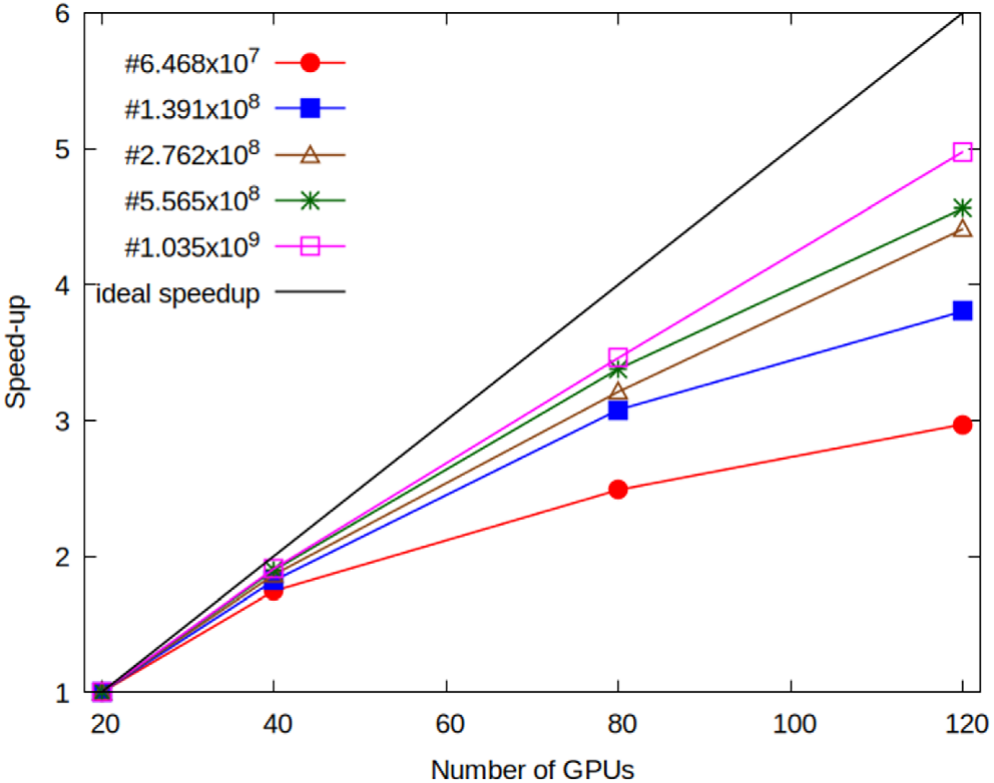
Strong scaling behavior of OCCAM code. Speed‐ups are calculated as the ratio t_5_/t between running times on five nodes (20 GPUs) and actual running times.

In order to have a more complete evaluation of the scalability of the proposed approach, the code performances are discussed in terms of strong and weak scaling. The scalability is related to how simulation time can change with the problem size and the number of GPUs. If the size of the simulated system is fixed and the number of GPUs increases, strong scaling is tested. The practical aim of strong scaling tests is to find a suitable setup that results in a reduction of simulation time by using a proper and reasonable amount of resources. Strong scaling tests, reported in Figure 12, indicate that convenient workloads should be not less than 1.5 million particles per GPU. Indeed, the speedup calculated for the smaller system (~65 million of particles), for the test using 40 GPUs (10 nodes), is close to the ideal value, while for the test using 80 GPUs (20 nodes) the calculated speedup is less than half of the ideal value. Similarly, for the system having ~139 million particles, the slope of the speedup curve decreases going from 80 to 120 GPUs where the workload approaches 1 million particles per GPU. For the three largest systems, larger workloads are tested (from 4 to 8 million of particles per GPU); in this case, larger slopes are always obtained.

Another type of scalability test is weak scaling. This test examines how the execution time changes when both the problem size and the number of computing units increase proportionally at constant workload. The aim is to maintain a constant workload per GPU while increasing the number of GPUs and the system size. If weak scaling is ideal, the execution time remains constant as the number of GPUs increases because each GPU handles the same amount of molecules. These tests help to determine if simulated systems can efficiently scale up, without performance degradation, to larger sizes as more computational resources are added. A summary of the results for weak scaling is reported in Table 4. According to the previously discussed strong scaling results, we tested systems having workloads always larger than 1.5 million particles per GPU. In particular, weak scaling tests have been performed at three different workloads. The system with the largest considered workload starts from 276 million particles on 20 GPUs (five nodes) and has a workload of ~14 million particles/GPU. Keeping the workload constant, test MD simulations have been performed using up to 80 GPUs (t20 nodes). On 20 nodes, the system size reaches more than 1 billion particles with a calculated efficiency of 0.87 quite close to the ideal value of one. This means that the execution time for the system scaled up (at constant workload per GPU) to 1 billion particles is only 15% longer than the one obtained for the smaller system. The second system with an intermediate workload (~7 million of particle/GPU) starting from 139 million particles on 20 GPUs has also been tested on 40 and 80 GPUs by reaching, on 20 nodes, a size of 557 million particles with a calculated weak scaling efficiency on 80 GPUs of 0.82 still close to ideal behavior. Finally, for the system with the smallest considered workload (~3 million of particle/GPU), reaching 276 million particles on 20 nodes, a satisfactory weak scaling efficiency of 0.66 has been calculated.

**TABLE 4:** Weak scaling performances calculated for systems of increasing size at constant workload (number of particles/GPU).

No. of nodes	Runtime^a^ (sec)	Weak scaling efficiency^b^ (*t*/*t*_5_)	Total number of particles ×10^8^	System
5	359.5	1	0.647	LB1
10	470.7	0.76	1.39	LB2
20	542.9	0.66	2.76	LB3
5	856.9	1	1.39	LB2
10	935.4	0.92	2.76	LB3
20	1038.9	0.82	5.57	LB4
5	1743.3	1	2.76	LB3
10	1851.0	0.94	5.57	LB4
20	1993.8	0.87	10.4	LB5

*Note:* Lipid bilayer systems LB1 to LB5 are those described in Table 2. For each of the three sets of data reported in the table, weak scaling efficiency is calculated as the ratio between the actual runtime *t* and the runtime of the smaller system *t*_*5*_ calculated using five nodes (20 GPUs).

^a^ Runtime for 50.000 MD timesteps.

^b^ Ratio between execution time on five nodes and actual execution time.

The hPF‐MD method implemented in OCCAM is based on a coarse‐graining technique, and performances obtained with any other software performing classical MD, also with a comparable number of particles, come from a more fine‐grained description of the systems. However, a direct comparison of running times between systems having a comparable number of particles and treated using classical MD based on pair forces can give an idea about the advantages of the proposed implementation and possible practical applications. A benchmark reported for AMBER code on a V100 GPU for 1.067.095 atoms (NPT simulation of Satellite Tobacco Mosaic Virus using 4 fs timestep: STMV, [60]) gives a production of 29.4 ns/day corresponding to 7.3 × 10^6^ steps/day. For a comparable system reported in Table 1, having 1 × 10^6^ particles (with 18 × 10^5^ DPPC molecules and ~8 × 10^7^ water beads), the OCCAM code, on the same GPU type, can produce 13 × 10^6^ steps/day. The computational advantage, as known already for CPU architectures, is larger for larger systems running on more computational units [25]. In particular, a benchmark reported for GROMACS code of peptides in water (Pep‐h 12, [61]), running for 12 × 10^6^ atoms on 10 nodes each with four NVIDA A100 GPUs, can run 20 × 10^6^ steps/day [62]. For a comparable system of DPPC lipids and water (reported in Table 1), having a size of 10 × 10^6^ particles, running on four A100 GPUs, the OCCAM code produces 100 × 10^6^ steps/day on 10 nodes with the same type and number of GPU per node. It is worth noting that all computations in OCCAM are made using double precision; this allows obtaining thermodynamic properties from calculated forces (e.g., Thermodynamic Integration or similar techniques, see refs. [48, 49, 50]), while GROMACS uses single precision. To the best of our knowledge, benchmarks for systems of sizes 100 million–10 billion particles have not been reported for classical MD on multi‐GPU architectures. However, to figure out the performances on this scale, it can be considered that a production of ~21 × 10^6^ steps/day (similar to the one with GROMACS for the benchmark Pep‐h12) on 10 nodes can be achieved for a system more than five times larger (65 million of particles) on 10 nodes. Due to good weak scaling performances, by doubling the number of nodes, the size can be further increased to 276 million beads running on 20 nodes with similar performances (~14 × 10^6^ steps/day).

## 5 Conclusions

A parallelization strategy of hPF‐MD simulations suitable for multi‐node multi‐GPU hardware has been documented. The resulting code performs, after a single initialization step, all the computations only on GPUs (GPU resident). According to the hardware architecture, the proposed implementation involves three different layers of parallelization: the first one among different nodes, the second one to better distribute the load on different GPUs present on different computational nodes, and the last one to exploit the intrinsic parallelism inside each GPU. The nature of the hPF‐MD methodology, using density fields to calculate non‐bonded forces (the most expensive part of MD calculations), allows minimizing both the size and frequency of data exchange. Large‐scale benchmarks, obtained for system sizes ranging from several million to 10 billion beads, show impressive performances and scalability. Benchmark results highlight the practical feasibility of hPF‐MD simulations on multi‐node/multi‐GPU architectures for multi‐billion particle systems in systematic studies. In particular, differing from classical MD simulations, where simulations on this scale require almost full occupation of large supercomputer centers, benchmarks obtained using a moderate number of nodes (from 5 to 30) on a very large biomembrane patch (1.5 × 1.5 μm) and a monoatomic fluid system of 10 billion particles show good performances. The proposed implementation shows good strong and weak scaling performances also for large‐scale systems ranging from 100 million to 10 billion particles. This opens the possibility to perform systematic/routine studies of large‐scale systems using a moderate quantity of computational resources and to reveal new molecular insights for problems previously inaccessible to molecular simulations.

## Conflicts of Interest

The authors declare no conflicts of interest.

## Supporting information


**Data S1.** Supporting Information.

## Data Availability

The data that support the findings of this study are available from the corresponding author upon reasonable request.
