# Supplementary material for: GPU Accelerated Hybrid Particle‐Field Molecular Dynamics: Multi‐Node/Multi‐GPU Implementation and Large‐Scale Benchmarks of the OCCAM Code
Source: J Comput Chem. 2025 May 14;46(13):e70126. doi: 10.1002/jcc.70126 (PMC12076535; doi:10.1002/jcc.70126)
Supplement: Supplementary file 1 — Data S1. Supporting Information. [file JCC-46-0-s001.doc]

SUPPLEMENTARY INFORMATION

**GPU accelerated Hybrid Particle-Field Molecular Dynamics: Multi-node/Multi-GPU Implementation and Large-Scale Benchmarks of OCCAM Code**

Rosario Esposito,^1^ Giuseppe Mensitieri,^1^ You-Liang Zhou,^2^ Zhong-Yuan Lu,^2^ Ying Zhao,^3^ Toshihiro Kawakatsu,^4^ Giuseppe Milano^1^

*^1^University of Naples Federico II, Department of Chemical, Materials and Production Engineering, Piazzale V. Tecchio, 80, 80125 Napoli, Italy*

*^2^State Key Laboratory of Supramolecular Structure and Materials, College of Chemistry, Jilin University, Changchun, 130012, China*

*^3^School of Physics and Materials Engineering, Dalian Minzu University, Dalian, Liaoning, 116600, China*

*^4^Department of Physics, Tohoku University, Aramaki, Aoba, Sendai 980-8578, Japan*

1. **Description of Lipid Water Coarse-Grained Models**

Systems containing lipids and water, have a coarse-grained representation of the molecules reported in Figure S1 for the phospholipid dipalmitoylphosphatidylcholine (DPPC) based on a four-to-one mapping of non hydrogen atoms in every coarse-grained bead.


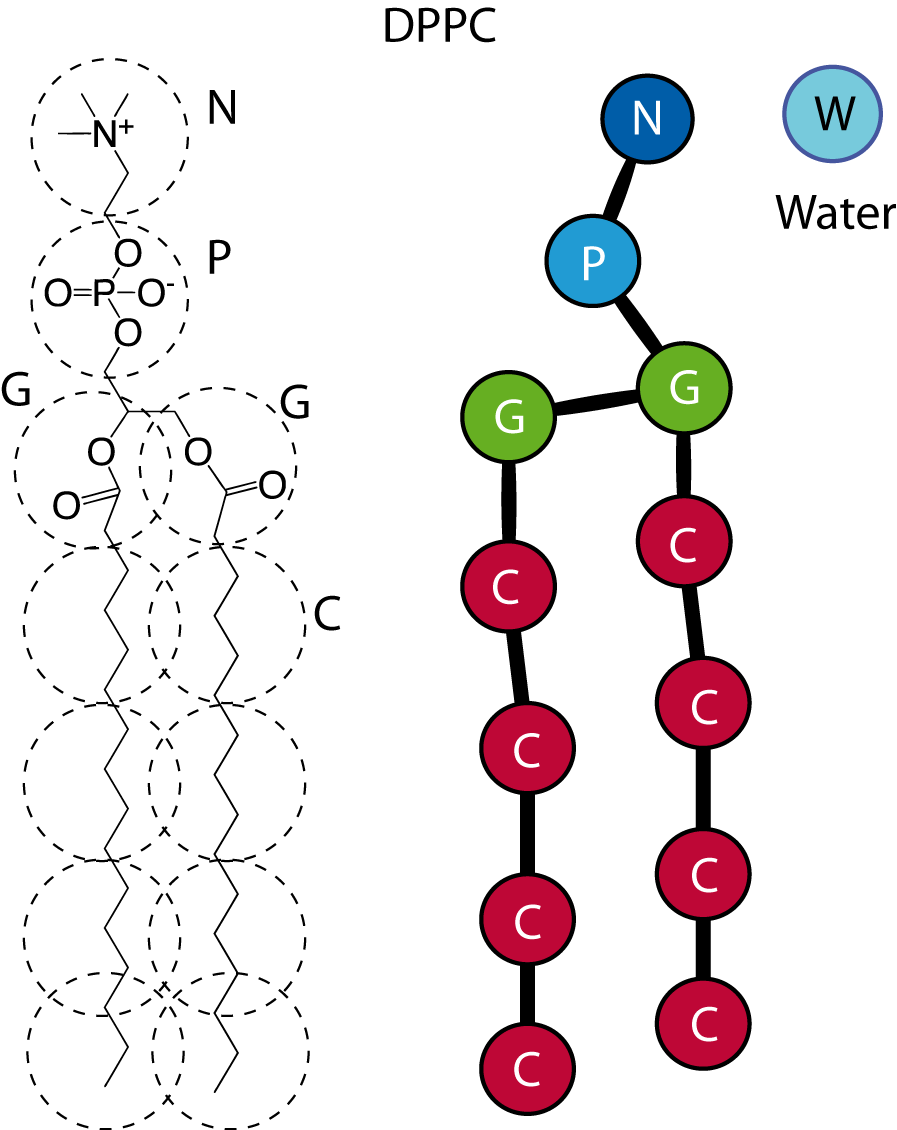


**Figure S1.** Mapping scheme for DPPC and water models used for benckmark calculations.

Bonds are described by harmonic potentials *V_bond_*(*R*) of the type

$V_{bond}(R)=\frac{1}{2}K_{bond}\left(R-R_{bond}\right)^{2}$ (S1)

where *R_bond_* is the equilibrium distance and *K_bond_*  is the force constant of the bond. The stiffness of the chains is taken into account by harmonic potentials *V_angle_*(*θ*) depending of the cosine of angle between atoms, where *θ*  is the angle between two successive bonds:

$V_{angle}(\theta)=\frac{1}{2}K_{angle}\left\{\cos(\theta)-\cos(\theta_{0})\right\}^{2}$ (S2)

where *K_angle_* is the force constant and *θ_0_* is the equilibrium bond angle. A water molecule is modeled as one particle. Particle-Field interactions parameters *χ_KK_*’ are fixed for every type of interaction between a particle *K* and the density field obtained from the particles of type *K*’. In the model considered, these parameters have been optimized to reproduce the properties of lipid bilayers from reference particle-particle simulations. Full list of parameters is reported in reference 1.

1. **Reduction Summation vs. Atomic Functions: Accuracy**


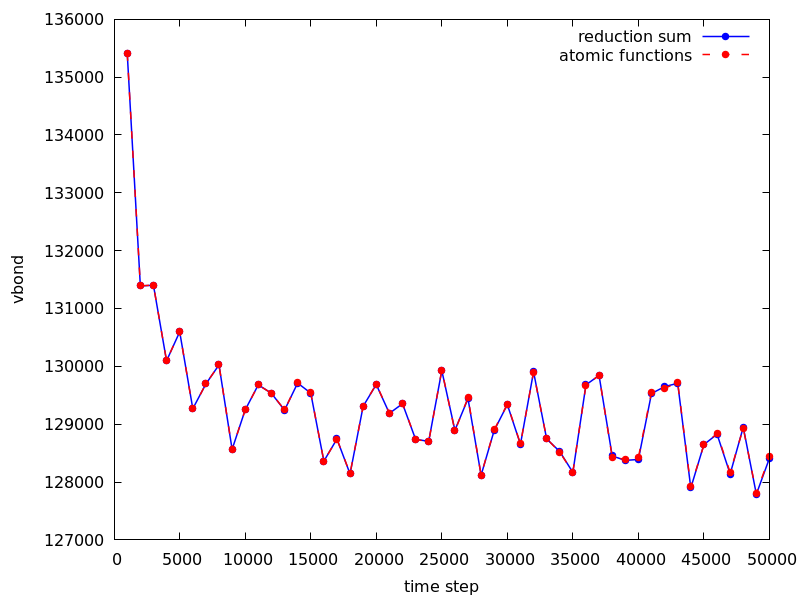


**Figure S2.** Comparison between bond potentials (sum over all bonds in the simulated system) obtained using atomicadd function (red curve) and reduction summation (blue curve).


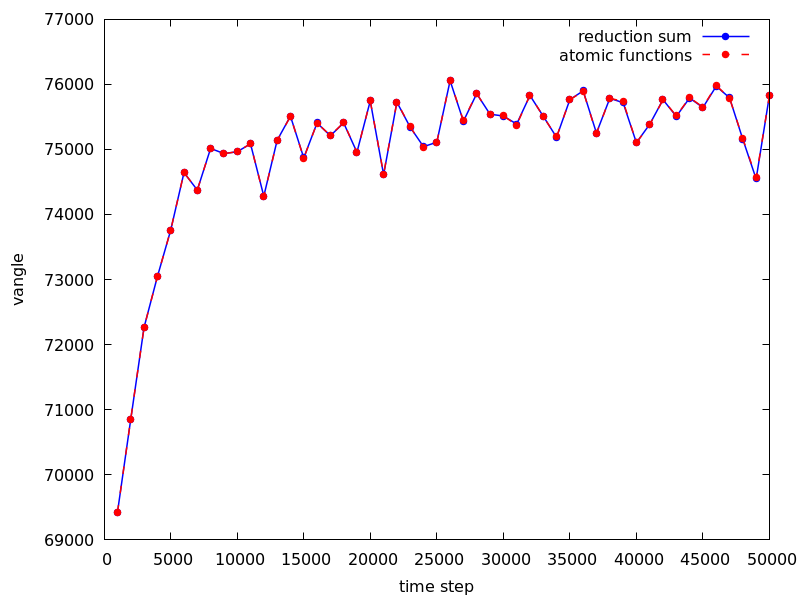


**Figure S3.** Comparison between angle potentials (sum over all angles in the simulated system) obtained using atomicadd function (red curve) and reduction summation (blue curve).

1. **Saddle Point Approximation**

The formulation of hPF-MD is routed in Self Consistent Field Theory (SCFT). Here we sketch the main ideas of SCFT and we will describe how to employ it to introduce the field representation of a molecular systems and for the decoupling of the related multibody problem and its typical use as starting point for the Mean Field Approximation (MFA). The total Hamiltonian of the *M*-molecule system can be split into two parts:

$\hat{H}\left(\Gamma\right)=\hat{H}_{0}\left(\Gamma\right)+\hat{W}\left(\Gamma\right)$ (A1).

Where Γ denotes the phase space point.

Assuming the canonical ensemble, where temperature, volume and number of particles are fixed, the configurational integral of this system is given by:

$Z=\frac{1}{M!}\int d\Gamma\exp\left\{-\beta\left[\hat{H}_{0}\left(\Gamma\right)+\hat{W}\left(\Gamma\right)\right]\right\},$(A1.2)

$\hat{H}_{0}\left(\Gamma\right)$ is the reference potential containing intramolecular interaction terms such as bond, angle, torsion etc. $\hat{W}\left(\Gamma\right)$is the non-bonded interaction. An important definition to connect particle and field models is the definition of molecular configuration as a microscopic density field:

, (A1.3)

where $\mathbf{r}_{i}^{(p)}$ denotes the position of particle *i* in the molecule *p*. By introducing the auxiliary fields $\psi\left(\mathbf{r}\right)$ and $\xi\left(\mathbf{r}\right)$it is possible to transform (A1.2) in the following way:

$Z=\int D\left\{\psi\left(\mathbf{r}\right)\right\}D\left\{\xi\left(\mathbf{r}\right)\right\}\exp\left\{-\beta\left[-\frac{1}{\beta}\ln Z\left[\xi\left(\mathbf{r}\right)\right]-\frac{i}{\beta}\int\xi\left(\mathbf{r}\right)\psi\left(\mathbf{r}\right)d\mathbf{r}+W\left[\psi\left(\mathbf{r}\right)\right]\right]\right\}$ (A1.4)

*D{ψ*(**r**)*}* and *D{ξ*(**r**)*}* denote the functional integration over the auxiliary fields *ψ*(**r**) and *ξ*(**r**).

By using the definition $Z\left[\xi\left(\mathbf{r}\right)\right]$:

$Z\left[\xi\left(\mathbf{r}\right)\right]=\frac{1}{M!}\int d\Gamma\exp\left\{-\beta\left[\hat{H}_{0}\left(\Gamma\right)+\frac{1}{\beta}i\int\xi\left(\mathbf{r}\right)\sum_{p=1}^{M} \sum_{i=0}^{N\left(p\right)} \delta\left(\mathbf{r}-\mathbf{r}_{i}^{(p)}\right)d\mathbf{r}\right]\right\}$ (A1.5)

The full derivation of (A1.4) is not here reported and the reader can find it in reference [24] of the paper. It is worth noting that eq. (A1.4) is exact and describes the partition function *Z* of the multibody system as the one of single molecules in external fields. The MFA is obtained, starting from (A1.4) by exploiting the so-called saddle point approximation. This approximation is realized by replacing the sum over the canonical ensemble with a Gaussian integral around the most probable state that minimizes the argument of the exponential function on the right-hand side of (A1.4). The canonical probability distribution of finding the system with takes its maximum value at which minimizes the free energy functional . If the total number of degrees of freedom of the system is large enough, the canonical probability distribution peaks sharply around . The canonical probability distribution can thus be well approximated by a Gaussian distribution centered at .

Let us minimize the argument of the exponential of (A1.4) with respect to $\xi\left(\mathbf{r}\right)$

$\begin{matrix} \frac{\delta}{\delta\xi\left(\mathbf{r}\right)}\left[-\frac{1}{\beta}\ln Z\left[\xi\left(\mathbf{r}\right)\right]-\frac{i}{\beta}\int\xi\left(\mathbf{r}\right)\psi\left(\mathbf{r}\right)d\mathbf{r}+W\left[\psi\left(\mathbf{r}\right)\right]\right]=0 \\ -\frac{1}{\beta Z\left[\xi\left(\mathbf{r}\right)\right]}\frac{\delta Z\left[\xi\left(\mathbf{r}\right)\right]}{\delta\xi\left(\mathbf{r}\right)}-\frac{i}{\beta}\psi\left(\mathbf{r}\right)=0 \end{matrix}$(A1.6)

From the first line of (A.1,5)

(A1.7)

According to (A1.6), we obtain

(A1.8)

By minimizing the argument of the exponential function in (A1.4) with respect to $\psi\left(\mathbf{r}\right)$

$\begin{matrix} \frac{\delta}{\delta\psi\left(\mathbf{r}\right)}\left[-\frac{1}{\beta}\ln Z\left[\xi\left(\mathbf{r}\right)\right]-\frac{i}{\beta}\int\xi\left(\mathbf{r}\right)\psi\left(\mathbf{r}\right)d\mathbf{r}+W\left[\psi\left(\mathbf{r}\right)\right]\right]=0 \\ \frac{i}{\beta}\xi\left(\mathbf{r}\right)=\frac{\delta W\left[\psi\left(\mathbf{r}\right)\right]}{\delta\psi\left(\mathbf{r}\right)} \end{matrix}$ (A1.9)

From the result of (A1.8) and (A1.9) we can finally write:

(A1.10)

Where $V\left(\mathbf{r}\right)\equiv\frac{i}{\beta}\xi\left(\mathbf{r}\right)$.

In the frame of MFA, according to (A1.10), both auxiliary fields $\psi\left(\mathbf{r}\right)$ and $i\xi\left(\mathbf{r}\right)$become real valued and correspond to the coarse-grained density and to the functional derivative of the interaction energy, respectively. In this case, $\xi\left(\mathbf{r}\right)$will be a purely imaginary field $\xi\left(\mathbf{r}\right)=-i\beta V\left(\mathbf{r}\right)$. The external potential $V\left(\mathbf{r}\right)$can be calculated as functional derivative of the interaction energy functional once that its functional form is given.

**References**

1. De Nicola, A.; Zhao, Y.; Kawakatsu, T.; Milano, G. J Chem Theory Comput 2011, 7, 2947–2962.
